# Supplementary figures and images for: Profiles and outcomes in patients with COVID-19 admitted to wards of a French oncohematological hospital: A clustering approach
Source: PLoS One. 2021 May 19;16(5):e0250569. doi: 10.1371/journal.pone.0250569 (PMC8133400; doi:10.1371/journal.pone.0250569)

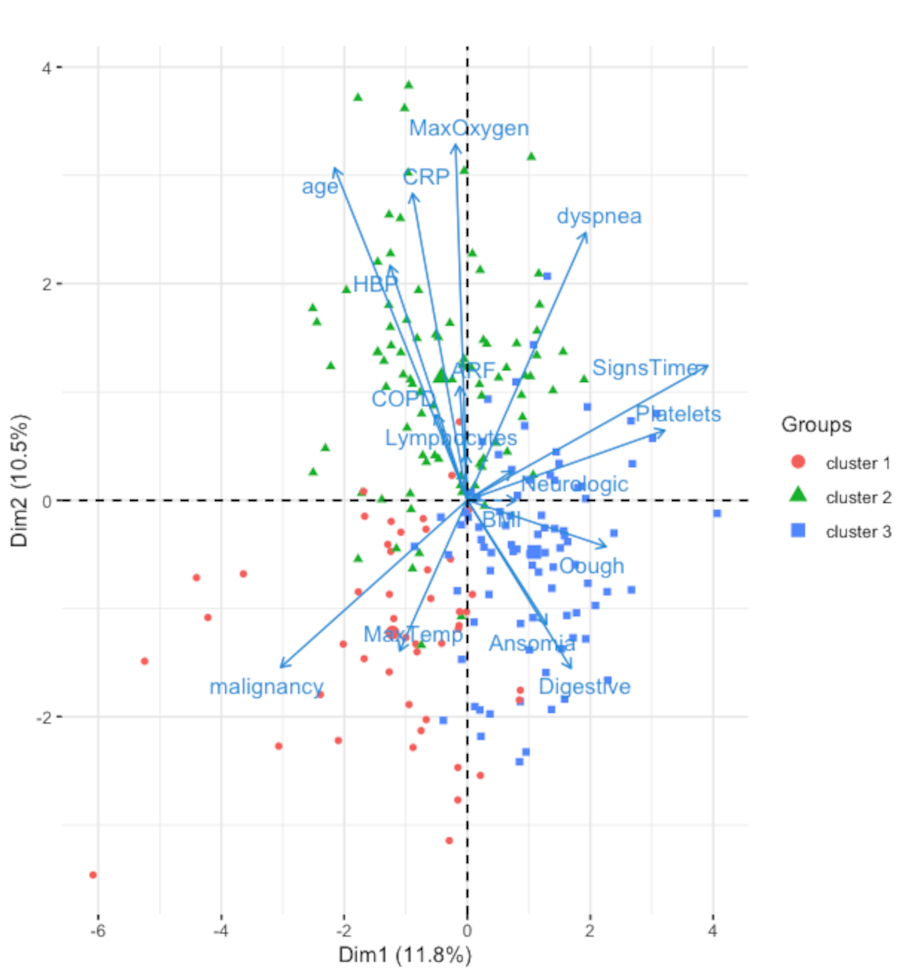

Supplement: S1 Fig — Representation of patients and variables (including anosmia) on the first two components of the PCA. COPD: chronic obstructive pulmonary disease, HBP: high blood pressure, Max oxygen: maximum oxygen flow over 24 h of admission, CRP: C-reactive protein, BMI: body mass index; Signs Time: time since disease onset. (TIF) [file pone.0250569.s001.tif]
